# Ankle proprioception correlates with functional mobility in people with Peripheral Neuropathy

**DOI:** 10.1186/1757-1146-7-S1-A54

**Published:** 2014-04-08

**Authors:** Shuqi Zhang, Li Li

**Affiliations:** 1 Department of Health and Kinesiology, Georgia Southern University, Statesboro, GA, 30458, USA; 2 School of Kinesiology, Louisianan State University, Baton Rouge, LA, 70803, USA

## Introduction

The ankle proprioception could influence the functional stability of ankle joint. In addition, ankle proprioception may indirectly influence postural control. Furthermore, ankle proprioception may play an important role in the impaired somatosensory system.

## Purpose

The purpose is to examine if ankle proprioception is correlated with functional mobility in people with Peripheral Neuropathy (PN) and health age-matched control.

## Methods

Twenty one people with, and twelve age-matched without PN, were recruited. Active (AAP) and passive (PAP) ankle proprioception were assessed using Biodex 3 dynamometer (Biodex Medical System, Inc, Shirley, NY, USA). Participants sat in the Biodex chair with the back of the chair positioned at 70° with lower leg parallel with the ground. The protocol of the active and passive reposition tests consisted of localizing three target positions: 15° of inversion, 0° subtalar neutral, 10° of eversion [1]. We have also tested foot sole sensation. The foot sole sensitivity (FSS) was tested at big toe (BT), 1st and 5th metatarsal (M1 and M5), midfoot (MF) and medial heel (MH) with a 5.07 monofilament [2]. The overall score of one foot was the number of its sensitive sites, ranged from 0 to 5. Functional mobility test (6-minute walk test and timed up-and-go test) were performed following standard procedures in both groups. Group effects were analyzed by ANOVA. Pearson correlation tests were used to examine the relationships between ankle proprioception tests and functional mobility measures.

## Results

There were significant different of AAP (PN: 28.2 ± 17.6, H: 16.8 ± 8.3), PAP (PN: 20.7 ± 12.6, H: 11.7 ± 4.3), FSS (PN: 2.5 ± 2.0, H: 4.3 ± 1.2), 6MW (PN: 426.9 ± 95.2, H: 525.3 ± 68.1), and TUG (PN: 9.7 ± 2.4, H: 6.5 ± 1.3) between two groups. No other significant group effect was observed among age, height and body mass. A significant positive correlation was observed between AAP /PAP and TUG in people with PN (R= 0.52, *P*<*.05*, R= 0.75, *P*<*.05*). A significant negative correlation was observed AAP/PAP and 6MW (R= -0.46, *P*<*.05*, R= -0.51, *P*<*.05*). No other significant correlation was observed.

## Discussion

Ankle proprioception is important for the functional mobility in the PN group, but not in the health control group. More accurate ankle proprioception correlates with faster walking speed in people with PN.
